# Association of ultra-processed foods consumption with increased liver steatosis in U.S. adults

**DOI:** 10.3389/fnut.2025.1536989

**Published:** 2025-03-13

**Authors:** Jingru Song, Siqi Chen, Kexin Qian, Wei Ye

**Affiliations:** ^1^Department of Gastroenterology, Hangzhou TCM Hospital of Zhejiang Chinese Medical University, Hangzhou, China; ^2^Zhejiang Chinese Medical University, Hangzhou, Zhejiang, China

**Keywords:** ultra-processed food, liver health, fatty liver disease, liver fibrosis, NHANES

## Abstract

**Background:**

Recent studies demonstrated a strong association between dietary habits and liver health, particularly in the development of steatosis and fibrosis. This study aimed to examine the impact of ultra-processed foods (UPFs) on liver health, focusing specifically on their influence on the risks of liver steatosis and fibrosis.

**Methods:**

A cross-sectional analysis was conducted on 4,992 participants aged 18 years and older from the 2017–2020 National Health and Nutrition Examination Survey (NHANES). Dietary intake was assessed using one or two 24-h dietary recalls, and foods were categorized by their processing level using the NOVA classification system. UPFs consumption was measured in grams and divided into quartiles. Liver health was assessed using controlled attenuation parameter (CAP) and liver stiffness measurement (LSM) via elastography, to evaluate steatosis and fibrosis, respectively. Linear regression models were applied to assess the relationship between UPFs consumption and liver outcomes, adjusting for sociodemographic (age, sex, ethnicity), lifestyle (alcohol consumption, physical activity), and biomedical factors (liver enzyme levels).

**Results:**

Higher UPF intake was significantly associated with increased CAP values, indicating a higher risk of liver steatosis. While liver fibrosis, measured by LSM, was also associated with UPF consumption, this relationship did not reach statistical significance. Multivariate analysis showed that increased UPF consumption did not significantly affect LSM (*p* = 0.110) but was strongly associated with elevated CAP values (*p* = 0.009). In participants with fatty liver (CAP > 248 dB/m), the association between UPF intake and CAP remained significant (*p* = 0.020). Participants in the highest quartile of UPFs consumption (Q4) exhibited higher CAP values compared to those in the lowest quartile (Q1) (β = 1.22; 95% CI: 1.02, 1.47). Stratified analysis revealed that the association between UPF intake and CAP was more pronounced in obese individuals (HR = 1.08, 95% CI: 1.03–1.15, *p* = 0.022) and those with high waist circumference (HR = 1.06, 95% CI: 1.01–1.10, *p* = 0.032).

**Conclusion:**

These results underscore the adverse impact of UPFs on liver health, particularly by increasing steatosis, while the connection with fibrosis remains less straightforward.

## 1 Background

Metabolic dysfunction-associated steatotic liver disease (MASLD) is rapidly emerging as a major global health concern, currently affecting ~32% of the adult population worldwide (1). Accounting for 59% of all chronic liver diseases (2), MASLD can progress to non-alcoholic steatohepatitis (NASH), significantly increasing the risks of cirrhosis, hepatocellular carcinoma (HCC), and mortality. The hallmark of MASLD is hepatic steatosis, characterized by excessive fat accumulation in the liver, which can lead to varying degrees of inflammation and fibrosis. This condition adversely affects metabolic, immune, and cardiovascular health, and is associated with an increased risk of hyperlipidemia and type 2 diabetes (3). A direct correlation was observed between the severity of hepatic steatosis and fibrosis progression (4), along with an increase in liver-related mortality (5). Dietary habits, particularly the consumption of soft drinks, red meat, and processed meats, are linked to an increased risk of MASLD, while diets low in free sugars—such as the Mediterranean diet—and those rich in dietary antioxidants may help reduce hepatic fat accumulation (6).

Ultra-processed foods (UPFs), characterized by their high content of refined ingredients and various additives, are typically lacking in whole food components. These products are often high in sugars, trans fats, sodium, and refined starches, yet deficient in essential nutrients such as fiber, protein, vitamins, and minerals (7). Numerous studies demonstrated a strong association between regular UPFs consumption and an increased risk of obesity in both children and adults (8, 9), as well as a higher prevalence of metabolic disorders, cardiovascular diseases (9–11), and certain cancers. From 2001 to 2018, UPFs consumption among American adults increased significantly, while intake of minimally processed foods declined (12). This dietary shift aligns with rising trends in obesity and metabolic syndrome in the United States, suggesting a potential connection between UPFs consumption and these growing health concerns. However, the specific relationship between UPFs intake and conditions such as fatty liver or liver fibrosis, particularly among adults, remains underexplored.

This study aims to investigate the association between UPF consumption and the prevalence of fatty liver and liver fibrosis in adults using data from the National Health and Nutrition Examination Survey (NHANES). By analyzing dietary patterns in a large, nationally representative adult sample, we seek to elucidate the potential role of UPFs in liver health and contribute to the growing body of research on the relationship between diet and liver disease.

## 2 Methods

### 2.1 Study participant

This investigation utilized NHANES dataset, a comprehensive series of cross-sectional surveys administered by National Center for Health Statistics (NCHS) under the auspices of the centers for disease control and prevention (CDC) (13). The NHANES protocol received approval from the NCHS Institutional Review Board, ensuring all participants provided written informed consent (14). Since its inception in 1999, NHANES has consistently enrolled around 6,000 individuals each year and continues to do so, with findings being disseminated biennially (15). Our analysis specifically targeted the 2017–2020 NHANES cohort, a period which included the acquisition of controlled attenuation parameter (CAP) and liver stiffness measurement (LSM) through vibration-controlled transient elastography (VCTE).

The study focused on adults aged 18 and above, who had complete LSM data and provided dual 24-h dietary recall information. These participants were selected via a sophisticated multistage probability sampling methodology. Initial data collection commenced with in-home interviews where participants completed a screener questionnaire. This was followed by structured interviews at mobile examination center (MEC) to assess eligibility based on detailed sociodemographic and health history. The MEC visits also included comprehensive physical examinations, laboratory testing, and dietary assessments. A follow-up dietary interview, conducted via telephone 3–10 days post-MEC visit, enabled the collection of in-depth dietary information from selected individuals. This rigorous process facilitated a detailed estimation of the type and quantity of food and beverage intake, encompassing their energy and nutrient profiles, as elaborated in the NHANES Dietary Interviewers Procedures Manual (16).

**Figure 1:**
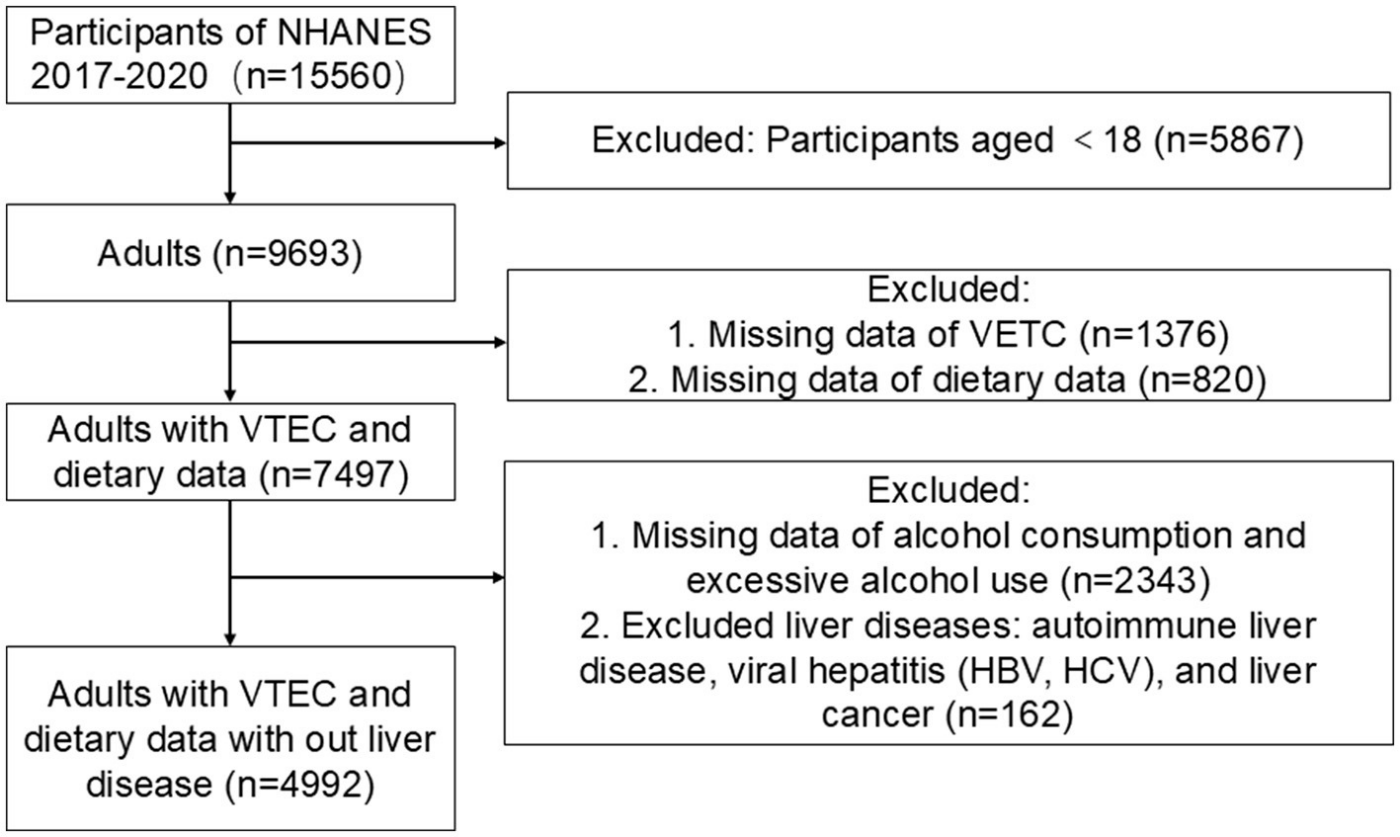
Flowchart of the study.

From the NHANES 2017−2020 data set, an initial pool of 15,560 individuals was considered. After excluding minors (*n* = 5,867), 9,693 adults were identified as potential participants. This number was further narrowed down by removing individuals with incomplete VCTE (*n* = 1,376) and dietary data (*n* = 820), leaving 7,497 subjects. In addition, participants with incomplete alcohol consumption data and those with excessive alcohol intake [5 or more alcoholic drinks (male), or 4 or more drinks (female), on the same occasion on at least 1 day in the past 30 days (17), *n* = 2,343] were excluded. Furthermore, individuals diagnosed with chronic liver diseases, including autoimmune liver disease, hepatitis B, hepatitis C, and liver cancer, were also excluded, resulting in a final analytical sample of 4,992 participants. This rigorous selection process ensured a robust sample representative of the adult population, facilitating an in-depth analysis of the relationship between UPFs consumption and health outcomes (Figure 1).

### 2.2 Dietary assessment

In this study, dietary intake data obtained from recalls were classified according to the NOVA system, which categorized foods based on their processing level (18). The NOVA system divides foods into four categories: unprocessed or minimally processed foods, processed culinary ingredients, processed foods, and UPFs (8).

Processed foods, like canned fish, vegetables, artisanal bread, and cheese, result from adding culinary ingredients to unprocessed foods. UPFs, on the other hand, are characterized by their industrial formulation and typically consist of five or more ingredients (19).

To assign foods or beverages to one of the four new categories, we employed the food codes provided by NHANES. For homemade recipes, NOVA was applied to the basic ingredients (standard reference codes). The USDA's Food and Nutrient Database for Dietary Studies for the specific period was used, with the USDA's National Nutrient Database serving as the standard reference (20). The food descriptions and ingredient lists for each NHANES food code were assessed against these databases.

### 2.3 VCTE evaluation of hepatic steatosis and fibrosis

Hepatic steatosis and fibrosis were assessed using VCTE via FibroScan. Measurements followed NHANES protocols for accuracy and reliability.

For evaluating liver fibrosis and steatosis, an LSM value exceeding 7 kPa was indicative of a high fibrosis risk (21). Steatosis was determined using a CAP threshold of over 248 dB/m (22).

### 2.4 Assessment of other variables

Demographic and lifestyle data were systematically gathered using computer-assisted personal interviewing (CAPI) system (23). Demographic information encompassed age, delineated into three categories (18–44, 45–59, and 60+ years) (24), and gender. Ethnic backgrounds were categorized into Hispanic, non-Hispanic White, non-Hispanic Black, non-Hispanic Asian, and Other/Multi-Racial (25). Educational attainment was classified as high school completion or higher. Marital status was segmented into married/living with partner and other classifications. Economic status was gauged through the poverty income ratio (PIR), separating individuals into low and non-low income groups.

Lifestyle variables assessed comprised smoking status, identified as current, former, or never smoker, and alcohol consumption, categorized into less than once a week, once a week or more, and abstinent in the past year. Physical activity was quantified based on self-reported instances of moderate and vigorous exercise. Body mass index (BMI) calculations were performed using height and weight measurements, conducted by trained professionals, with BMI computed as the individual's weight in kilograms divided by the square of their height in meters, rounded to one decimal point. BMI was categorized using a cutoff of 25 kg/m^2^, classifying individuals as lean or obese (13). Waist circumference was stratified based on sex-specific thresholds, with high waist circumference defined as ≥102 cm for men and ≥88 cm for women (26).

Biological markers pertinent to liver health were selectively included based on their presence in NHANES data and relevance in scientific literature. These markers included alanine aminotransferase (ALT), aspartate aminotransferase (AST), and gamma-glutamyl transferase (GGT), providing a comprehensive overview of potential liver function abnormalities.

### 2.5 Statistical analysis

Categorical and continuous variables were characterized using frequencies (*n*), percentages (%), and quartiles, respectively. Due to the skewed nature of the data, the χ∧2 test was employed for categorical variables, and the Kruskal-Wallis test was applied to continuous variables for comparative analyses. Multivariate logistic regression models were utilized to calculate odds ratios (ORs) and 95% confidence intervals (CIs) for liver steatosis (defined as CAP > 248 dB/m) and significant fibrosis (LSM > 7 kPa) across the quartiles of UPFs consumption (Q1 through Q4).

The analysis included covariates that could potentially affect liver fibrosis and steatosis, such as age, ethnicity, education level, marital status, annual household income, BMI, waist circumference, alcohol and smoking status, physical activity, and liver enzyme levels. To evaluate potential differences in the association between UPF consumption and hepatic steatosis across subgroups, we conducted stratified analyses. Stratification was performed based on age, sex, BMI, and waist circumference to explore potential effect modification.

To ensure that the study findings were representative of the U.S. population, survey sample parameters, including clustering, strata, and weights, were meticulously integrated into the statistical analysis. All analyses were performed using R version 4.3.3 (R Core Team, Vienna, Austria), with statistical significance set at a *p*-value of < 0.05 (two-tailed).

## 3 Results

### 3.1 Clinical characteristics of the study participants

Referencing Table 1 from the NHANES 2017–2020 dataset, which included 4,992 adults, demographic analysis revealed statistically significant disparities in gender and ethnic distributions across UPFs consumption quartiles. Notably, a higher proportion of males, non-Hispanic White participants was observed in Q4. With an increase in UPFs consumption, BMI and waist circumference increased, while physical activity declined. These observations underscored a significant link between UPFs consumption and liver health metrics, where elevated CAP values (*p* < 0.001) in the highest quartile hinted at an increased risk for steatosis. This pattern indicates a tangible correlation between dietary habits and health outcomes, particularly in the context of liver fibrosis and steatosis.

**Table 1:** Characteristics by categories of UPFs among adults NHANES 2017–2020.

**Characteristic**	**Overall, *N* =4,922 (100%)^a^**	**Q1, *N* = 1,511(25%)^a^**	**Q2, *N* = 1,292 (25%)^a^**	**Q3, *N* = 1,177 (25%)^a^**	**Q4, *N* = 1,012 (25%)^a^**	***P*-Value^b^**
Gender						**< 0.001**
Female	2,592 (52%)	961 (66%)	742 (60%)	570 (51%)	319 (32%)	
Male	2,400 (48%)	550 (34%)	550 (40%)	607 (49%)	693 (68%)	
Age (year)						0.130
18–44	2,164 (46%)	649 (48%)	561 (47%)	517 (45%)	437 (43%)	
45–59	1,227 (26%)	349 (24%)	305 (23%)	290 (26%)	283 (31%)	
≥60	1,601 (28%)	513 (29%)	426 (30%)	370 (28%)	292 (27%)	
Race						**< 0.001**
Hispanic	1,011 (15%)	349 (19%)	285 (17%)	226 (14%)	151 (9.3%)	
Non-Hispanic Asian	608 (6.3%)	360 (16%)	129 (5.1%)	85 (3.3%)	34 (1.3%)	
Non-Hispanic Black	1,353 (12%)	395 (13%)	394 (14%)	340 (12%)	224 (7.6%)	
Non-Hispanic White	1,776 (63%)	343 (49%)	427 (61%)	471 (67%)	535 (77%)	
Other/multi-racial	244 (3.9%)	64 (3.8%)	57 (3.7%)	55 (3.7%)	68 (4.5%)	
Education						**0.010**
High education	4,061 (93%)	1,152 (91%)	1,052 (92%)	1,011 (95%)	846 (92%)	
Low education	648 (7.5%)	240 (9.5%)	156 (7.8%)	115 (5.1%)	137 (7.5%)	
Marital						**0.013**
Married/living with partner	2,798 (61%)	810 (57%)	686 (57%)	695 (64%)	607 (66%)	
Other	2,194 (39%)	701 (43%)	606 (43%)	482 (36%)	405 (34%)	
PIR						0.300
Low income	725 (11%)	233 (12%)	180 (11%)	169 (9.1%)	143 (11%)	
Not low income	3,655 (89%)	1,072 (88%)	945 (89%)	880 (91%)	758 (89%)	
Alcohol						**< 0.001**
No drinking in the past year	683 (9.8%)	377 (19%)	157 (11%)	95 (5.5%)	54 (3.7%)	
Less than once a week	2,801 (54%)	812 (54%)	788 (59%)	669 (56%)	532 (48%)	
Once a week or more	1,506 (36%)	321 (27%)	347 (30%)	413 (39%)	425 (48%)	
Missing	2 (< 0.1%)	1 (< 0.1%)	0 (0%)	0 (0%)	1 (< 0.1%)	
Smoking						**< 0.001**
Current smoker	711 (13%)	115 (8.2%)	143 (10%)	194 (14%)	259 (22%)	
Former smoker	1,069 (23%)	238 (16%)	271 (22%)	302 (30%)	258 (25%)	
Never smoker	3,211 (63%)	1,157 (76%)	878 (68%)	681 (57%)	495 (53%)	
BMI (kg/m^2^)	28 (24, 33)	27 (23, 31)	28 (24, 33)	29 (25, 33)	29 (26, 34)	**< 0.001**
Waist circumference (cm)	98 (87, 110)	93 (83, 104)	97 (86, 108)	99 (89, 112)	102 (91, 113)	**< 0.001**
Vigorous activity						**0.002**
Yes	1,458 (33%)	457 (39%)	407 (37%)	343 (31%)	251 (26%)	
No	3,534 (67%)	1,054 (61%)	885 (63%)	834 (69%)	761 (74%)	
Moderate activity						0.200
Yes	2,235 (51%)	726 (55%)	590 (53%)	498 (48%)	421 (47%)	
No	2,755 (49%)	785 (45%)	701 (47%)	678 (52%)	591 (53%)	
ALT (U/L)	18 (13, 27)	17 (13, 24)	18 (14, 27)	17 (13, 26)	20 (15, 30)	**< 0.001**
AST (U/L)	19 (16, 23)	19 (16, 23)	20 (16, 24)	19 (16, 23)	19 (16, 24)	0.140
GGT (U/L)	19 (13, 30)	17 (12, 27)	19 (13, 30)	19 (14, 28)	22 (15, 36)	**< 0.001**
LSM (kPa)	4.80 (4.00, 6.00)	4.70 (3.80, 5.70)	4.80 (4.00, 5.90)	4.90 (4.00, 6.20)	5.00 (4.20, 6.20)	**0.003**
CAP (dB/m)	256 (215, 304)	240 (208, 291)	249 (211, 295)	256 (212, 306)	275 (231, 319)	**< 0.001**

^a^Median (IQR) for continuous; *n* (%) for categorical.

^b^Chi-squared test with Rao & Scott's second-order correction; Wilcoxon rank-sum test for complex survey samples.

The values in bold indicate *P* < 0.05.

### 3.2 Multivariate analysis of factors influencing factors on MASLD and hepatic fibrosis relative to UFP quartiles

Table 2 examines the correlation between UPFs consumption and liver health parameters within the NHANES 2017–2020 adult cohort, specifically analyzing LSM and CAP across UPFs consumption quartiles. In the unadjusted model, higher UPF intake was significantly associated with increased LSM values, showing a positive trend (*p* for trend < 0.001). After adjusting for demographic factors (Model 1), the association remained significant, particularly in the highest UPF quartile (Q4: β = 1.15; 95% CI: 1.08–1.22). However, when further adjusting for lifestyle factors such as alcohol consumption, smoking status, and waist circumference (Model 2), the association attenuated and became non-significant (*p for* trend = 0.140). In the fully adjusted model (Model 3), which included biochemical markers (ALT, AST, and GGT), the association between UPF consumption and LSM remained statistically insignificant (*p* for trend = 0.110), suggesting that UPF intake may have a limited impact on liver fibrosis.

**Table 2:** Relationship between UPFs and VCTE in adults in NHANES.

	**Non-adjusted model**		**Model 1**		**Model 2**		**Model 3**	
	β	**95% CI**	β	**95% CI**	β	**95% CI**	β	**95% CI**
**LSM (kPa)(continues)**								
Q1	Ref.		Ref.		Ref.		Ref.	
Q2	1.34	1.00, 1.78	1.07	1.00, 1.14	1.03	0.96, 1.10	1.02	0.94, 1.09
Q3	2.04	1.40, 2.97	1.13	1.05, 1.22	1.07	0.99, 1.15	1.07	0.97, 1.17
Q4	2.52	1.71, 3.73	1.15	1.08, 1.22	1.05	0.99, 1.12	1.06	0.99, 1.14
*P* for trend	**< 0.001**		**< 0.001**		0.140		0.110	
**CAP (dB/m) (continues)**								
Q1			Ref.		Ref.		Ref.	
Q2	1.03	1.00, 1.07	1.05	1.00, 1.09	1.02	0.99, 1.05	1.02	0.98, 1.05
Q3	1.06	1.03, 1.10	1.05	1.01, 1.09	1.01	0.91, 1.04	1.02	0.98, 1.05
Q4	1.12	1.09, 1.15	1.10	1.05, 1.15	1.04	1.01,1.07	1.04	1.00 1.08
*P* for trend	**< 0.001**		**< 0.001**		**0.003**		**0.009**	

Model 1: Adjusted for demographic factors (gender, age, race, education level, marital status).

Model 2: Further adjusted for lifestyle factors (alcohol intake, smoking status, waist circumference).

Model 3: Further adjusted for biochemical markers (ALT, AST, GGT).

The values in bold indicate *P* < 0.05.

Table 2 highlights that increased UPF intake is significantly associated with higher CAP values, suggesting a greater likelihood of hepatic steatosis. Higher UPF intake was strongly associated with increased CAP values, indicating a higher risk of liver steatosis. The unadjusted model showed a significant association (*p* for trend < 0.001), with CAP values increasing across UPF quartiles. This association remained robust in Model 1 after adjusting for demographic factors (Q4: β = 1.10; 95% CI: 1.05–1.15). Even after further adjustments for lifestyle factors in Model 2 and biochemical markers in Model 3, the association persisted (Model 3: Q4: β = 1.04; 95% CI: 1.00–1.08; *p* for trend = 0.009).

Table 3 in the results segment presents the link between UPFs intake and liver health indicators. It provides beta coefficients and 95% CIs across UPFs consumption quartiles for LSM (>7 kPa) and CAP (>248 dB/m), which serve as fatty liver and fibrosis, respectively. The unadjusted model showed a significant association (*p* < 0.001) between increased UPFs consumption and elevated LSM and CAP values. After adjusting for demographic, lifestyle, and metabolic factors, this association remained significant, particularly in the highest UPF quartile. However, in Models 2 and 3, the p-trend for LSM was no longer statistically significant, while the association with CAP remained robust. These findings suggested that excessive UPF consumption was independently associated with an increased risk of hepatic steatosis, underscoring the potential impact of dietary patterns on liver health.

**Table 3:** Association of UPFs with fatty liver and liver fibrosis in adults in NHANES.

	**Non-adjusted model**		**Model 1**		**Model 2**		**Model 3**	
	β	**95% CI**	β	**95% CI**	β	**95% CI**	β	**95% CI**
**LSM** >**7 kPa**								
Q1	Ref.		Ref.		Ref.		Ref.	
Q2	1.00	0.98, 1.03	1.21	0.74, 1.97	1.03	0.63, 1.69	1.05	0.60, 1.84
Q3	1.04	1.01, 1.08	1.69	1.03, 2.78	1.26	0.76, 2.10	1.21	0.67, 2.17
Q4	1.05	1.02, 1.08	1.75	1.10, 2.80	1.19	0.71, 2.00	1.27	0.68, 2.36
*P* for trend	**0.002**		**0.031**		0.6		0.7	
**CAP** > **248 dB/m**								
Q1	Ref.		Ref.		Ref.		Ref.	
Q2	1.11	0.95, 1.30	1.17	0.97, 1.40	1.06	0.88, 1.29	1.06	0.85, 1.33
Q3	1.29	1.09, 1.52	1.24	1.02, 1.52	1.06	0.91, 1.25	1.09	0.89, 1.33
Q4	1.56	1.35, 1.81	1.48	1.21, 1.81	1.21	1.04,1.41	1.22	1.02, 1.47
*P* for trend	**< 0.001**		**< 0.001**		**0.022**		**0.020**	

Model 1: Adjusted for demographic factors (gender, age, race, education level, marital status).

Model 2: Further adjusted for lifestyle factors (alcohol intake, smoking status, waist circumference).

Model 3: Further adjusted for biochemical markers (ALT, AST, GGT).

The values in bold indicate *P* < 0.05.

### 3.3 Dose-response analysis of UPFS with CAP and LSM values

**Figure 2:**
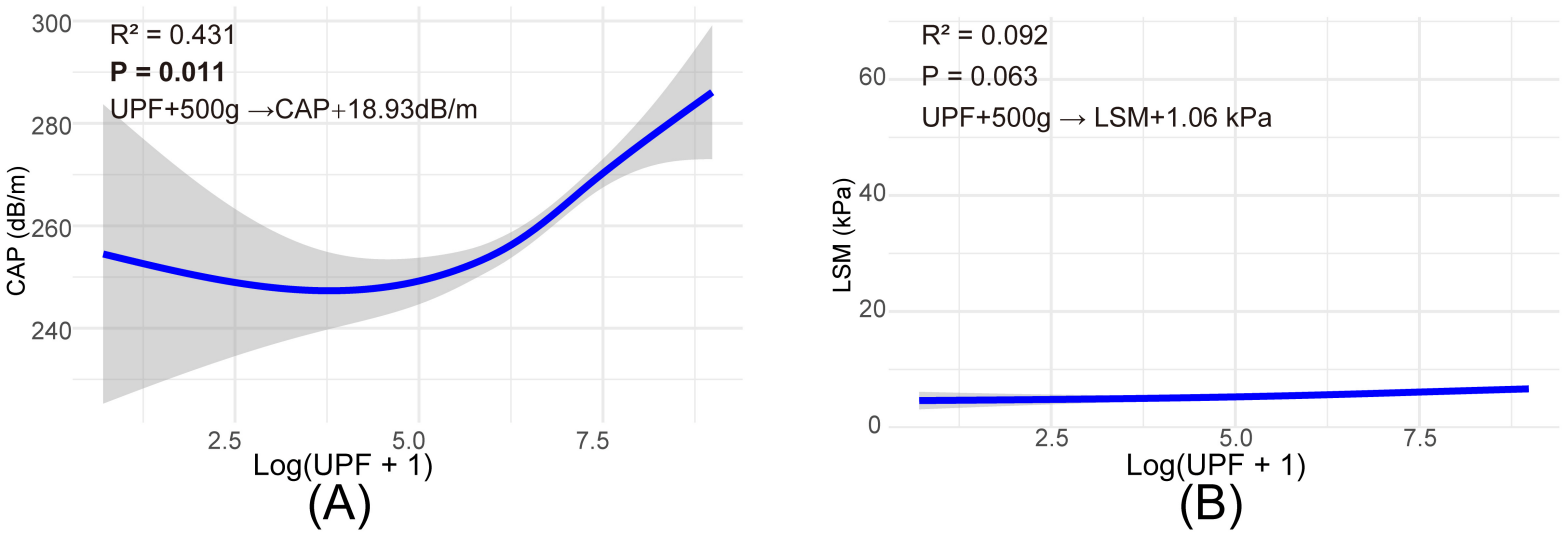
Smooth curve fitting to describe the linear relationship between log(UPF + 1) consumption and VTEC. **(A)** Association between log(UPF +1) consumption and CAP values, **(B)** association between log(UPF +1) consumption and LSM values.

Figure 2 illustrates a graphical insight into the relationship between UPFs consumption and liver health metrics derived from VCTE in healthy adults. Panel A depicts the correlation between UPFs intake and the CAP, expressed on a log-transformed scale (log(UPF + 1)), indicative of fatty liver deposition. The scatter plot in this panel shows an upward trend, with smooth curve fitting indicating that increased UPFs consumption is associated with higher CAP values, signaling enhanced liver fat accumulation. The 95% confidence interval, represented by the shaded area, underscores the statistical reliability of this trend. Panel B presents the relationship between UPFs consumption, expressed on a log-transformed scale (log(UPF + 1)), and LSM, a biomarker for liver fibrosis. The graphical representation here shows a relatively constant LSM value across different levels of UPFs intake, as denoted by the nearly flat line. These visual analyses highlight that UPFs exerted a more pronounced effect on liver fat accumulation than on liver stiffness across the analyzed UPFs consumption spectrum. The graphical representation facilitates the comprehension of the potential dietary influences on liver health metrics, with a statistically significant impact observed in CAP trends (*p* < 0.05), contrasting with the non-significant trends in LSM. Additionally, a quantitative analysis revealed that an increase of 500 g/day in UPF consumption corresponded to an estimated 18.93 dB/m increase in CAP but had a more modest effect on LSM (1.06 kPa increase).

### 3.4 Subgroup analyses

**Figure 3:**
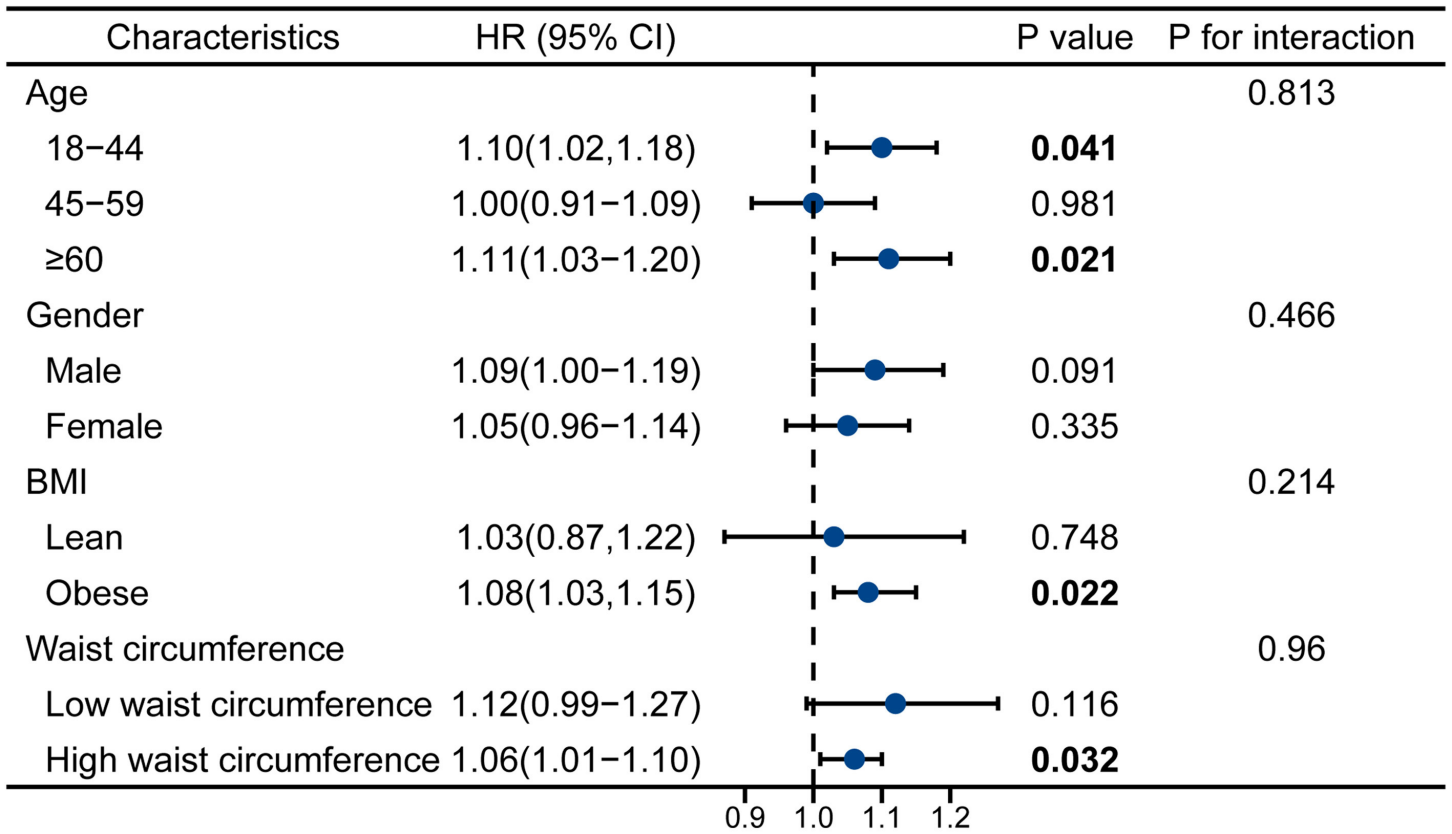
Stratified analysis of UPF consumption and CAP across different subgroups.

Figure 3 presents a stratified analysis of the association between UPF consumption and CAP, further elucidating its impact across different subgroups. The results indicate that the association between UPF intake and CAP remains consistent across multiple demographic and metabolic subgroups, with higher UPF consumption corresponding to increased CAP values. Notably, the effect of UPFs on CAP was more pronounced among individuals with obesity and those with high waist circumference, suggesting a potential interaction between excess adiposity and dietary patterns in hepatic fat accumulation. These findings reinforced the independent association between UPF intake and liver fat deposition while highlighting the modifying effects of metabolic risk factors.

## 4 Discussion

In this cross-sectional analysis of 4,992 American adults, we found that increased UPFs consumption is linked to a higher risk of developing fatty liver and liver fibrosis, as evidenced by the accelerated accumulation of liver fat.

Socio-demographic factors played a significant role in UPFs consumption patterns, with higher intake predominantly seen in males, non-Hispanic White, and regular alcohol consumers. This indicates varied dietary habits across different demographic groups. Historical NHANES data from 1999 to 2010 indicated that the dietary quality of non-Hispanic White adults was generally lower than that of Mexican American adults (25). Additionally, a rise in UPFs consumption correlated with an increase in BMI, highlighting the substantial influence of UPFs on the prevalence of overweight and obesity. In line with prior research, our analysis also demonstrated a positive relationship between UPFs consumption and body fat accumulation (25).

Our study establishes a definitive link between UPFs consumption and increased liver fat content, leading to a higher risk of fatty liver disease. Individuals with greater UPFs intake showed significant increases in liver fat. Utilizing CAP with a cutoff of > 248 dB/m for fatty liver definition, these individuals had a considerably elevated risk. In a detailed analysis of a subgroup of adults with obesity and metabolic syndrome, a higher consumption of UPFs was consistently linked to increased visceral fat, an elevated fat ratio, and greater total body fat accumulation (27). The consumption of saturated fats is known to quickly increase liver lipid storage, alter energy metabolism and insulin resistance, and affect liver gene expression and signaling pathways, potentially accelerating the onset of fatty liver disease (28). Studies showed that diets low in carbohydrates and fats, combined with aerobic and resistance exercises, led to reductions in body weight, total and visceral fat, and hepatic lipid content (29), ultimately decreasing liver fat (30).

Our examination of the connection between UPFs consumption and liver fat buildup considered multiple factors. UPFs often have a poor nutritional profile, enriched with high levels of saturated and trans fatty acids to enhance flavor and stability (7), factors closely linked to increased liver fat in humans. Additionally, UPFs typically lack dietary fiber (31), a deficiency tied to the development of MASLD. Large-scale studies demonstrate an inverse relationship between dietary fiber intake and MASLD prevalence (32). Dietary fiber is vital for maintaining gut microbiome balance and increasing satiety, which indirectly reduces the intake of high-fat and high-sugar foods, thereby lowering the risk of liver fat accumulation. UPFs are also rich in refined carbohydrates, leading to postprandial hyperglycemia (33), closely associated with disturbances in glucose, insulin, and lipid metabolism, crucial factors in liver fat increase (34, 35). Furthermore, experimental studies show that certain additives in UPFs, like nanoparticles, can induce gastotoxicity and hepatotoxicity, and disrupt the gut microbiome (36), highlighting the complex risks of UPFs consumption and its potential impact on liver health.

The normal liver parenchyma, supported by thin connective tissue capsules and the extracellular matrix (ECM), maintains flexibility, allowing increased blood flow without significant intrahepatic pressure rise. However, an increase in ECM components, especially collagen, and subsequent changes in liver parenchyma vascular architecture lead to increased tissue stiffness. Fibrosis involves a significant rise in fibrous tissue or collagen, directly associated with increased tissue stiffness (37). Liver fibrosis is a dynamic condition where excessive ECM buildup, prompted by injury and inflammation, is balanced by its degradation and remodeling (38). When fibrogenesis surpasses degradation, it alters vascular structures, leading to cirrhosis. This fibrosis progression is often slow initially, potentially accelerating in later stages or under immunocompromised conditions. LSM aligns with liver fibrosis stages, showing gradual increases in early disease phases (stages 0–2) and sharp rises in advanced stages (stages 3–4) (39). Our study used LSM to examine the effect of UPFs consumption on liver fibrosis and found that although LSM values increased with higher UPFs intake, the rise wasn't consistent, preventing a definitive claim that increased UPFs consumption directly heightens liver fibrosis risk. This variability may be due to the slow progression of fibrogenesis in early fibrosis, affected by factors like inflammation, edema, venous congestion, and biliary obstruction, which all increase liver parenchyma stiffness. Moreover, the specific nutritional content of different UPFs categories could differently influence fibrogenesis, making it challenging to establish a direct causal link between UPFs consumption and fibrosis risk.

Our study boasts significant strengths, such as its large, nationally representative American sample, lending external validity to our findings. Using LSM and CAP as biomarkers provides accurate, objective liver health assessments. Nevertheless, the study's cross-sectional nature limits our ability to deduce temporal causality. Confirming our results requires longitudinal studies. Additionally, daily food consumption variability and potential dietary recall bias, possibly leading to UPFs intake underreporting, need careful consideration. The varied impact of different UPFs categories on liver health also requires further detailed study. Prospective research is crucial to validate our findings. If confirmed, reducing UPFs consumption could become a key strategy for preserving liver health in adults.

## 5 Conclusions

In conclusion, our research emphasizes a strong correlation between UPFs consumption and the risk of fatty liver disease in American adults, with a higher intake of UPFs associated with increased liver fat. The association between UPFs consumption and liver fibrosis, however, is less clear, necessitating further study to clarify the mechanisms and potential causal links. Prospective studies are needed to confirm these findings and assess the long-term effects of UPFs on liver health. Limiting UPFs intake may be a strategic preventive measure against fatty liver disease and fibrosis, thus improving liver health outcomes in the adult population.

## Data Availability

The original contributions presented in the study are included in the article/supplementary material, further inquiries can be directed to the corresponding author.
